# Type 2 Diabetes and Myocardial Infarction: Recent Clinical Evidence and Perspective

**DOI:** 10.3389/fcvm.2021.644189

**Published:** 2021-02-24

**Authors:** Jing Cui, Yanfei Liu, Yiwen Li, Fengqin Xu, Yue Liu

**Affiliations:** ^1^Cardiovascular Centre of Xiyuan Hospital, China Academy of Chinese Medical Sciences, Beijing, China; ^2^National Clinical Research Centre for Chinese Medicine Cardiology, Beijing, China; ^3^Institute of Clinical Pharmacology, Xiyuan Hospital of China Academy of Chinese Medical Sciences, Beijing, China; ^4^China Center for Evidence-Based Medicine of Chinese Medicine, Beijing, China

**Keywords:** type 2 diabetes, myocardial infarction, evidence-based medicine, drug, sodium-glucose cotransporter-2 inhibitor

## Abstract

Type 2 diabetes mellitus (T2DM) and its complications are seriously affecting public health worldwide. Myocardial infarction (MI) is the primary cause of death in patients with T2DM. T2DM patients without a history of coronary artery disease (CAD) have the same risk of major coronary events as those with CAD; T2DM patients with a history of MI have >40% risk of recurrence of MI. Thus, CAD in patients with T2DM needs to be treated actively to reduce the risk of MI. The cardiology community focused on the role of T2DM in the development of CAD and on the related issues of T2DM and MI with respect to comorbidities, prognosis, drug therapy, and heredity. In this mini review, the latest progress of clinical evidence-based research between T2DM and MI in recent years was reviewed, and the possible research directions in this field were considered and prospected.

## Introduction

Type 2 diabetes mellitus (T2DM) is one of the leading chronic non-communicable disease, and its prevalence has significantly increased globally. In 2017, the prevalence of adult T2DM accounted for 8.8% of the world population, and this proportion is expected to increase to 9.9% by 2045 (1, 2). With the increasing number of cases, T2DM and its complications are seriously affecting the quality of human life and have become a serious global public health problem. A pooled analysis of 22 prospective cohort studies encompasses more than one million subjects in Asia found that Asian patients with T2DM are at a higher risk of death than patients from the Western countries, with an 89% increase in mortality compared with that of those without T2DM (3). China has become a “hardest hit area” by T2DM; among Chinese adults, the estimated overall prevalence of T2DM is 10.9%, and the prevalence of pre-T2DM is 35.7% (4). Myocardial infarction (MI) is the primary cause of death in T2DM patients, and the risk of major coronary events in T2DM patients without a history of coronary artery disease (CAD) is equal to that in patients with CAD, with the >20% risk of a first MI within 10 years of developing T2DM, which is equal to the risk of a second MI within 10 years in non-T2DM patients with a history of MI, while the risk of recurrence of MI in the future in T2DM patients with MI history exceeds 40% (5). In a scientific statement published in *Circulation* on April 13, 2020 (6), the American Heart Association (AHA) noted that compared with CAD in patients without T2DM, CAD in patients with T2DM needs to be treated more aggressively to reduce the risk of MI. Although cardiologists have been treating patients with CAD and associated T2DM for a long time, T2DM has traditionally been considered a comorbidity that only affects the development and progression of the CAD. In the past decade, many factors have changed, forcing the cardiology community to reconsider the important role of T2DM in the development and progression of CAD. In addition to being associated with increased cardiovascular (CV) risk, T2DM may influence the choice of multiple treatments for CAD. Thus, glycemic control is recommended as part of the comprehensive risk factor management for patients with CAD; there has been growing evidence that the mechanisms of glycemic control have a significant impact on CV outcomes (7).

With the publication of the results of several large clinical trials on oral hypoglycemic drugs with CV benefits in recent years, people are more concerned about the comorbidities, prognosis, drug treatment, genetics, and other issues related to T2DM and MI.

In this review article, by mainly retrieving PubMed, MEDLINE, EMBASE, and Web of Science, we identified and critically analyzed nearly 5 years (from January 1, 2016, until December 30, 2020) of published clinical studies [randomized controlled trials (RCTs) and cohort studies] focusing on T2DM and MI. The search terms were “diabetes, type 2 diabetes mellitus, myocardial infarction, MI, cardiovascular disease, cardiovascular safety, cardiovascular events, cardiovascular risk, cardiovascular outcomes.” The retrieval formula was appropriately adapted to different databases.

In this paper, the latest progress in evidence-based clinical research on T2DM and MI in recent years has been reviewed, and the possible research directions in this field in the future have been considered and prospected.

## Comorbid Features of Type 2 Diabetes Mellitus and Myocardial Infarction

In 2019, the *Guidelines on Diabetes, Pre-Diabetes and Cardiovascular Diseases* jointly issued by the European Society of Cardiology (ESC) and European Association for the Study of Diabetes (EASD) recommends that all patients with cardiovascular disease should be screened for T2DM and that patients with cardiovascular disease complicated by T2DM should undergo comprehensive risk factor management, including control of blood pressure, serum glucose, and lipid levels; management of antiplatelet therapy regimens; and lifestyle interventions (8). The trends of mortality and morbidity in MI patients after 1 year suggested that long-term trends in survival and CV outcomes have improved considerably in patients with MI; however, their risk of mortality and morbidity in MI remains higher than that of the general population, especially when additional risk factors such as T2DM, hypertension, or advanced age are present (9, 10).

The prevalence of unrecognized abnormal glucose tolerance (AGT) and the incidence of recurrent CV events in patients with MI have not been systematically assessed. A meta-analysis of the prevalence of AGT in MI patients without a history of DM as well as the risk of recurrent major adverse cardiac events (MACEs) and mortality in MI patients was conducted. In the 19 clinical studies included (*n* = 541,509 with a median follow-up of 3.1 years), the prevalence of newly discovered AGT in patients with MI was 48.4%. Patients with prediabetes had a higher risk of death and MACE than did patients with normal glucose tolerance (NGT). Newly diagnosed T2DM cases showed a higher risk of death and MACE occurrence than NGT cases (11). Clinical research on the prevalence and prognosis of MI in asymptomatic T2DM patients has also been conducted to determine the prevalence of unrecognized MI in asymptomatic T2DM patients using delayed-enhancement MRI (DE-MRI), and the results of up to 5 years of follow-up in 460 T2DM patients showed that the incidence of death or MI was significantly higher in unidentified T2DM patients and that unidentified MI was prevalent in asymptomatic T2DM patients without a history of heart disease (12).

Researchers are increasingly concerned about the relationship between prediabetes and the risk of CV disease (CVD) and mortality (13–15). A meta-analysis of the association between prediabetes and the risk of CVD and mortality including 129 studies with a total of 10,069,955 patients showed that prediabetes increased risk of all-cause mortality and CVD and that prevention of prediabetes was important for patients with CVD (16).

A systematic review and meta-analysis of the correlation between T2DM and long-term (≥1 year) post-MI mortality was conducted, including 10 RCTs and 56 cohort studies (714,780 patients), with a total of 202,411 deaths over a median follow-up time of 2.0 (range, 1–20) years; it was found that the high long-term mortality of patients with T2DM was significant over time, independent of the phenotype of MI and modern treatments, and the long-term mortality was approximately 50% higher in patients with T2DM than in those without T2DM (17). Patients with T2DM had worse short- and long-term prognoses than those without T2DM, and undiagnosed T2DM was significantly correlated with higher mortality, especially in patients still with undiagnosed T2DM at the time of hospital admission (5).

Intracoronary drug-eluting stent (DES) percutaneous coronary intervention (PCI) is currently one of the standard treatments for patients with acute coronary syndrome (ACS), including those with MI, and T2DM also has a negative impact on the treatment and outcome of patients after PCI. Early-stage arterial healing after DES-PCI makes short-term dual antiplatelet therapy (DAPT) possible. A study (18) used coronary angiography [coronary artery stenosis (CAS)] data to compare the intravascular status of T2DM patients (*n* = 149) and non-T2DM patients (*n* = 188) in the early post-DES-PCI period, and it found that 3–5 months after DES implantation, DM patients showed more uncovered stent wires than non-DM patients, suggesting that the recent ultrashort DAPT strategy may not be applicable to patients with concomitant T2DM. Meanwhile, new-onset T2DM [new onset of DM (NODM)] after DES-PCI is receiving increasing attention. A study (19) used a retrospective cohort design to report the incidence, predictors, and long-term clinical outcomes of NODM after DES-PCI in patients with MI. The study reviewed 6,048 patients after PCI, grouped according to the presence or absence of T2DM before PCI, and found that 436 (11.8%) of 3,683 patients with ACS who did not have a diagnosis of T2DM before PCI had developed NODM over the 3.4 ± 1.9 years of follow-up, with independent predictors including high-dose statin therapy, high body mass index (BMI), and high fasting plasma glucose (FPG) and triglyceride levels. The cumulative MACE rate over 8 years of follow-up were significantly lower in the group with NODM after PCI (19.5%) than in the group with preoperatively diagnosed T2DM (25%, *P* = 0.003) and comparable with the group without T2DM (20.5%, *P* = 0.467). A retrospective cohort study conducted in Taiwan that included a larger number of patients (20) (30,665 patients diagnosed with ACS undergoing PCI) found a significant 27% increased risk of NODM in patients using statins than in those not using statins. The benefits of statins in preventing morbidity and mortality in patients with ACS have been validated in several clinical trials, and the clinical decision to recommend statin therapy for patients with pre-existing CVD should not be altered.

Knowledge about the mechanisms responsible for diabetes accelerating MI has increased enormously in recent years. The mechanisms by which hyperglycemia and insulin resistance increased mortality after MI were increasingly understood (21). Most diabetic patients are complicated with insulin resistance, hyperinsulinemia, and vascular calcification, which not only promote the occurrence of atherosclerosis but also accelerate the progression of stable plaques to unstable plaques or plaque rupture leading to thrombosis, thus leading to the occurrence of coronary adverse events (22). Contributing factors [including diabetes-induced overexpression of reactive oxygen species (ROS); secretion of inflammatory cytokines; increased aldose reductase (AKR1B1) substrate conversion; and activation of protein kinase C β, δ, and θ] accelerate the occurrence of MI (21).

Not only is T2DM strongly associated with MI, but its complications are also closely related with MI. A systematic review and meta-analysis of cohort studies on association between diabetic retinopathy (DR) and CVD included a total of 13 studies representing 17,611 patients, which suggested that DR is remarkably related with increased risk of CVD and CVD-associated mortality in diabetes (23). In addition, a hospital-based cross-sectional study in China included 949 patients (700 males and 249 females) with T2DM both non-proliferative DR (NPDR) and proliferative DR (PDR) independently associated with increased cardio-ankle vascular index (CAVI) (24). Other studies have also shown that central atherosclerosis is associated with the presence and severity of DR in patients with T2DM (25).

It is generally acknowledged that T2DM is an independent risk factor of acute kidney injury (AKI). Meanwhile, AKI predicts poor prognosis in patients with MI. The data from a multicenter factorial RCT included 10,251 participants who showed an incremental graded risk for CVD outcomes and all-cause mortality with the development of chronic kidney disease (CKD) and/or CVD in individuals with T2DM (26). Experimental evidence suggests that treatment with the sodium-glucose cotransporter-2 (SGLT2) inhibitor protects the diabetic kidney from MI-induced AKI (27).

## Shift From “Cardiovascular Safety” to “Cardiorenal Benefit” of Oral Hypoglycemic Agents

The discovery and clinical application of insulin as well as the subsequent introduction of various oral hypoglycemic agents (OHAs) have greatly prolonged the survival time of T2DM patients and transformed T2DM into a major chronic non-communicable disease. The effect of CV-related complications on the prognosis of patients with T2DM has attracted more attention and finally became the primary problem to be solved in improving the clinical prognosis of patients with T2DM. Few large-scale CV outcome trials (CVOTs) have been conducted to verify the CV safety of traditional OHAs. Since 2007, when rosiglitazone was found to significantly increase the risk of MI in T2DM patients (28), the CV safety of OHAs has received more attention. In 2008, the US Food and Drug Administration (FDA) developed a guidance document (29) to clarify the CV safety of innovative OHAs for the treatment of T2DM, which requires clinical evaluation of CV safety for all agents used for the treatment of T2DM prior to marketing. Therefore, CVOTs have been conducted for all OHAs marketed after 2008 to evaluate their CV safety.

**Figure 1:**
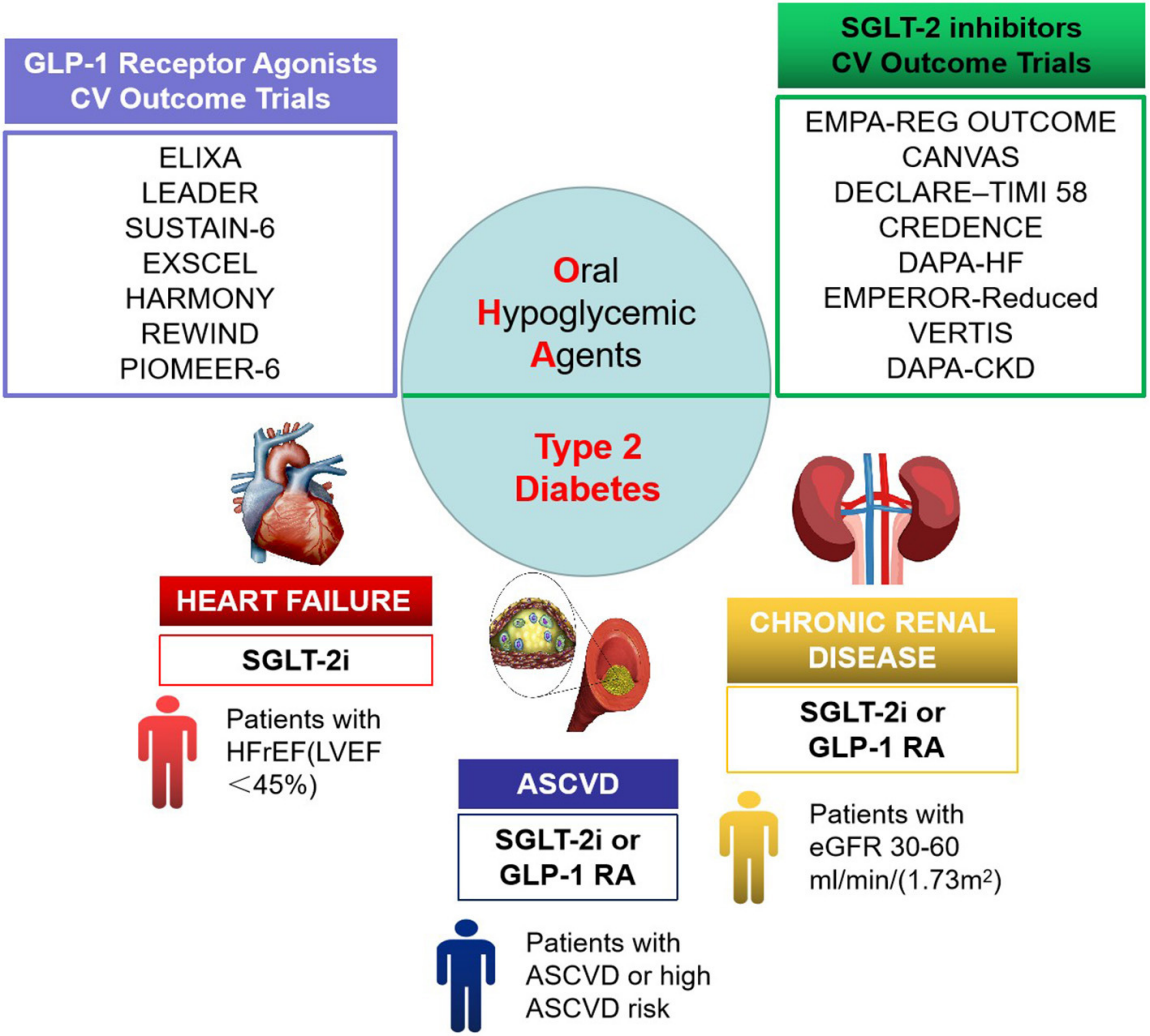
Cardiorenal benefit of oral hypoglycemic agents in therapeutic focus of type 2 diabetes mellitus (T2DM).

SGLT2 inhibitor is an innovative oral hypoglycemic drug marketed in recent years, and its main mechanism of action is to lower blood glucose by inhibiting the reabsorption of glucose by the renal proximal convoluted tubules and promoting urinary glucose excretion. In 2015, the results of the EMPA-REG OUTCOME trial (30, 31) were published, making empagliflozin the first oral hypoglycemic drug with definite CV benefits. In T2DM patients with comorbid CVDs, empagliflozin significantly reduced the risk of three-point MACE (3P-MACE) by 14%, and the risk of CV death and hospitalization for heart failure (HF) by 38 and 35%, respectively. Over the past 5 years, results from several large clinical trials on SGLT2 inhibitors have been published (30, 32–38) (see Table 1), and the expectation for OHAs has gradually changed from “cardiovascular safety” to “cardiovascular benefits.” Among the renal outcome studies, EMPA-REG OUTCOME was the first to report a 39% reduction in the risk of developing a renal composite endpoint with empagliflozin, suggesting that empagliflozin may delay the progression of renal diseases (39). A clinical study evaluating the effect of canagliflozin on renal events of patients with DM complicated by renal diseases with renal outcomes as a primary endpoint also demonstrated that canagliflozin reduced the risk of composite endpoints by up to 30% (34). The therapeutic focus of T2DM has also changed, from an exclusive focus on glucose-lowering parameters, to comprehensive management, and then to the current therapeutic focus on cardiac benefits and renal outcomes (see Figure 1).

**Table 1:** Characteristics of the RCT studies about SGLT2 inhibitors.

**Clinical trials**	**EMPA-REG outcome (30)**	**CANVAS (32)**	**DECLARE–TIMI 58 (33)**	**Credence (34)**	**DAPA-HF (35)**	**Emperor-reduced (36)**	**Vertis (37)**	**DAPA-CKD (38)**
ClinicalTrials.gov number	NCT01131676	NCT01032629/NCT01989754	NCT01730534	NCT02065791	NCT03036124	NCT03057977	NCT01986881	NCT03036150
Year	2015	2017	2019	2019	2019	2020	2020	2020
Participants (*n*)	7,020	10,142	17,160	4,401	4,744	3,730	8,246	4,304
Age, years (mean)	63.1	63	63.9	63	66	67	64	62
Men (%)	71.2	64.9	63.1	66	77	76	70	67
SGLT2 inhibitor agent	Empagliflozin	Canagliflozin	Dapagliflozin	Canagliflozin	Dapagliflozin	Empagliflozin	Ertugliflozin	Dapagliflozin
Dose (mg·day^−1^)	10, 25	100, 300	10	100	10	10	5, 15	10
Eligibility criteria	≥18 years of age; clinical diagnosis of type 2 diabetes; established cardiovascular disease, glycated hemoglobin level of 7~10%, eGFR ≥ 30 ml·min^−1^·(1.73 m^2^)^−1^	≥30 years of age with a history of symptomatic atherosclerotic cardiovascular disease or ≥50 years of age with two or more of the following risk factors for cardiovascular disease: clinical diagnosis of type 2 diabetes; glycated hemoglobin level 7.0~10.5%, eGFR ≥ 30 ml·min^−1^·(1.73 m^2^)^−1^	≥40 years of age; clinical diagnosis of type 2 diabetes; multiple risk factors for atherosclerotic cardiovascular disease or had established atherosclerotic cardiovascular disease glycated hemoglobin level 6.5~12%; creatinine clearance ≥ 60 ml·min^−1^	≥30 years of age; clinical diagnosis of type 2 diabetes; glycated hemoglobin level of 6.5–12.0%; eGFR 30~90 ml·min^−1^·(1.73 m^2^)^−1^; urine albumin: creatinine ratio 300~5,000 mg·g^−1^	≥18 years of age; New York Heart Association (NYHA) class II, III, or IV symptoms, ejection fraction ≤ 40%; NT-proBNP ≥ 600 pg·ml^−1^ (or ≥ 400 pg·ml^−1^ if they had been hospitalized for heart failure within the previous 12 months, or NT-proBNP ≥ 900 pg·ml^−1^ if they are with atrial fibrillation or atrial flutter on baseline electrocardiography regardless of their history of hospitalization for heart failure)	≥18 years of age; NYHA class II, III, or IV symptoms; ejection fraction ≤ 40%	≥40 years of age; clinical diagnosis of type 2 diabetes; established atherosclerotic cardiovascular disease involving the coronary, cerebrovascular, or peripheral arterial systems; glycated hemoglobin level of 7~10.5%	≥18 years of age, eGFR 25~75 ml·min^−1^·(1.73 m^2^)^−1^, urine albumin: creatinine ratio 200~5,000 mg·g^−1^
Follow-up, years (median)	3.1	2.4	4.2	2.6	1.5	1.3	3	2.4
Percentage of patients with confirmed cardiovascular disease at baseline (%)	99.4	71.2	40.5	50.4	100	100	76.1	37.4
Percentage of patients with heart failure at baseline (%)	9.9	14	9.9	15	100	100	24	11
Proportion of patients with eGFR ≥ 30 ml·min^−1^·(1.73 m^2^)^−1^ at baseline (%)	74.2	76.7	85.4	100	100	–	100	85.5

*Albumin-to-creatinine ratio was calculated with albumin measured in milligrams and creatinine measured in grams*.

*SGLT2, sodium-glucose cotransporter-2; eGFR, estimated glomerular filtration rate; NT-proBNP, N-terminal pro-B-type natriuretic peptide; RCT, randomized controlled trial*.

SGLT2 inhibitors exhibited superiority; thus, the cardiologist approve that SGLT2 inhibitors should be used in great property, at least in T2DM patients with high CV risk (40). What is more, major international guidelines all highly recommend the use (or combined use) of SGLT2 inhibitors in patients with T2DM with comorbid CVDs (or high risk of CVDs) and/or CKD, with the ESC/EASD guidelines (7) recommending them over metformin in newly diagnosed patients. The overall degree of recommendation by the American Association of Clinical Endocrinology/American College of Endocrinology (AACE/ACE) guidelines (41) is comparable with that for metformin.

Dipeptidyl peptidase-4 (DPP-4) inhibitors also occupy an increasing place in the management of T2DM. DPP-4 inhibitors may enhance homing of endothelial progenitor cells and thereby exert vascular protection (42). Available evidence suggests that DPP-4 inhibitors have a weak CV protective effect (43). However, in clinical application, it should be selected according to the actual situation. In patients with T2DM with advanced CVD or HF associated with renal function deterioration, DPP-4 inhibitors appear to be safe to use from a cardiological point of view, and SGLT2 inhibitors are contraindication (44). Of note, a study based on a large diabetic cohort of 113,051 T2DM patients showed that DPP-4 inhibitors as a second- or third-line add-on treatment provided CV benefits and posed no increased risks for HF, hypoglycemia, or death (45).

Glucagon-like peptide-1 receptor agonists (GLP-1 RAs) is viewed as the primary DPP-4 substrate capable of modulating CV function. The study found that use of GLP-1 RAs was associated with significant reductions in CV and all-cause mortality, and the researchers suggested that GLP-1 RAs should be used as a first-line treatment in patients with T2DM at higher CV risk or as a first-line treatment in patients with metformin resistance (46).

## Perspective

### Exploration of Early-Stage Screening Methods

The comprehensive management of T2DM patients is important for early identification and detection of possible CV risks. An effective risk prediction model was established by Chinese scholars, and it was found that weight reduction, lowering blood pressure and blood uric acid levels, and proper control of diastolic blood pressure could significantly reduce the risk of new-onset ACS in T2DM patients in northwest China (47). The meta-analysis included 30 studies with 253,425 participants and 1,621,920 person-years of follow-up, which is about prognosis of unrecognized MI determined by electrocardiography or cardiac MRI. Unrecognized myocardial infarction (UMI) by electrocardiography (ECG) or UMI by cardiac MR (CMR) is associated with an adverse long-term prognosis similar to that of recognized MI (48). Imaging evidence indicates a high prevalence of CAD in patients with T2DM; however, there is no standard for initiating CAD screening in the T2DM population, and it has been found that routine screening for CAD using computed tomographic coronary angiography (CTCA) should be considered for early detection of CAD in asymptomatic T2DM patients with diagnosed duration of T2DM >10.5 years and systolic blood pressure > 140 mmHg (49). A study (50) has established the triglyceride glucose index (TGI), calculated as [fasting triglycerides (mg·dl^−1^) × FPG (mg·dl^−1^)/2], to predict CV events and demonstrated that TGI may be a better predictor for the risk of CV events than FPG or glycosylated hemoglobin (HbA1c) in patients with ACS undergoing PCI. Statins have been widely used for lipid-lowering therapy in patients with CAD; however, there has been increasing clinical evidence of a correlation between statin use and NODM. A study (51) found that systolic epicardial adipose tissue thickness is an independent predictor for NODM in patients with CAD treated with high-intensity statins, which can help physicians formulate timely and appropriate monitoring or intervention plans for the early detection of NODM.

### Exploration From a Genetic Perspective

Scholars have conducted useful exploration on which T2DM patients are more likely to develop MI from a genetic perspective. The common MTNR1B single-nucleotide polymorphism locus rs10830963 is strongly correlated with the risk of developing T2DM. The relationship between this genetic variation and the risk of MI in patients with T2DM has been investigated using data from a UK Biobank cohort, which investigated the relationship between rs10830963 and the incidence of MI (fatal and non-fatal) in 13,655 participants with possible T2DM over a 6.8-year follow-up period. With the use of an additive genetic model, variation in the MTNR1B gene rs10830963 was found to be positively correlated with the risk of developing MI over 6.8 years of follow-up, suggesting that rs10830963 polymorphism may be a useful genetic marker for the development of MI in patients with T2DM (52). The growing knowledge of the genetic insights between T2D and CVD is beginning to provide the potential understanding of both disorders (53). In the future, the subtle relationship between T2DM and MI can be explored further deeply in terms of genetics.

### Diversity of Drug Evaluation Methods

In the clinical evaluation of the CV safety of innovative oral hypoglycemic drugs, although RCTs can better exclude the influences of confounding factors, they have poor external validity and cannot fully meet the actual clinical needs. Therefore, combining RCTs with real-world research/study (RWR/RWS) can provide more reliable and high-level clinical evidence, and real-world evidence (RWE) studies can be used as auxiliary evidence to RCTs to evaluate the efficacy and safety of drugs, which can help fill the knowledge gap between RCTs and actual clinical practice (54). RCTs provide evidence for clinical guideline recommendations, and real-world studies test the practicability of guideline recommendations, thus allowing step-by-step refinement of treatment strategies to optimize treatment and return to clinical practice. RWS includes a wider population to compensate for the poor external validity of RCTs due to stringent inclusion and exclusion criteria. A credible RWS covers a large number of patients and can reflect the routine clinical practice, which is of clinical significance for treatment and especially for safety assessment.

Moreover, SGLT2 inhibitors have been used to treat patients with T2DM to reduce the risk of CV events, including HF, and it is clear that the mechanism by which SGLT2 inhibitors reduce this risk may not be directly related to improved diabetic status and glycemic control. In addition, short-term use of SGLT2 inhibitors significantly improved volume load and symptoms in HF patients with concomitant T2DM; however, serum N-terminal pro-B-type natriuretic peptide (NT-proBNP) concentrations, which are traditionally used to assess HF severity, did not improve significantly, suggesting that we need to explore meaningful biomarkers that can monitor and evaluate the effect SGLT2 inhibitors for HF treatment in the future (55), in order to provide scientific evidence for in-depth understanding of the many unknowns of SGLT2 inhibitors.

## Author Contributions

JC and YaL formed the reference collection, conducted the reference analysis, and wrote the manuscript and are considered as co-first authors. YuL and FX contributed to the topic conception, manuscript revision, and decision to submit for publication and are the co-corresponding authors. YiL contributed to reference analysis and helped in the revision of the manuscript. All authors contributed to the article and approved the submitted version.

## Conflict of Interest

The authors declare that the research was conducted in the absence of any commercial or financial relationships that could be construed as a potential conflict of interest.
